# Advancing Medical Decision-Making with AI: A Comprehensive Exploration of the Evolution from Convolutional Neural Networks to Capsule Networks

**DOI:** 10.3390/jimaging12010017

**Published:** 2025-12-30

**Authors:** Ichrak Khoulqi, Zakariae El Ouazzani

**Affiliations:** 1 DICC Team, Data4Earth Laboratory, Sultan Moulay Slimane University, Beni Mellal 23000, Morocco; 2 Laboratory of Artificial Intelligence, Data Sciences and Emerging Systems, School of Applied Sciences, Sidi Mohamed Ben Abdellah University, Fes 30000, Morocco; zakariae.elouazzani2@usmba.ac.ma

**Keywords:** CNNs, CapsNets, segmentation, classification, medical decision-making

## Abstract

In this paper, we propose a literature review regarding two deep learning architectures, namely Convolutional Neural Networks (CNNs) and Capsule Networks (CapsNets), applied to medical images, in order to analyze them to help in medical decision support. CNNs demonstrate their capacity in the medical diagnostic field; however, their reliability decreases when there is slight spatial variability, which can affect diagnosis, especially since the anatomical structure of the human body can differ from one patient to another. In contrast, CapsNets encode not only feature activation but also spatial relationships, hence improving the reliability and stability of model generalization. This paper proposes a structured comparison by reviewing studies published from 2018 to 2025 across major databases, including IEEE Xplore, ScienceDirect, SpringerLink, and MDPI. The applications in the reviewed papers are based on the benchmark datasets BraTS, INbreast, ISIC, and COVIDx. This paper review compares the core architectural principles, performance, and interpretability of both architectures. To conclude the paper, we underline the complementary roles of these two architectures in medical decision-making and propose future directions toward hybrid, explainable, and computationally efficient deep learning systems for real clinical environments, thereby increasing survival rates by helping prevent diseases at an early stage.

## 1. Introduction

Biomedical image processing typically starts with the conversion of raw pixels into useful visual information. Fundamental operations, such as convolution filtering, edge or texture detection, and gradient computation, assist in revealing local contrast information and features necessary for later-stage diagnosis. The variables used during this early processing have been known to assist in detection, even with slight variation in terms of the system capacity to extract information necessary for diagnosis [1,2,3]. These developments have been further accelerated by machine learning (ML) and deep learning (DL), which enable algorithms to extract useful features directly from data without relying on human-designed descriptors. Nonetheless, even with these developments, models used for medical imaging are vulnerable to several issues: these models are heavily dependent on large and heterogeneous datasets, respond poorly to noise or imaging artifacts, and typically do not deal with patient or imaging device variability. Such issues are responsible for the challenges in constructing good models for healthcare [4]. CNNs have proven to be the backbone of various medical imaging analyses. These methods are useful for segmentation, classification, and detection. The hierarchical structure facilitates automatic representation learning in CNNs. CNNs enhance the accuracy of computer-aided diagnosis, disease models, and identification systems separately [1,2,3]. CNNs have limitations in situations where correct spatial information is required. It can be noted that because of the pooling layers, which greatly decrease dimensions by discarding position information, CNNs are sensitive to translation and rotation variability; hence, they do not work well in complex medical imaging problems [4]. CapsNets seem to manifest themselves as a necessary architectural answer to these deficits and limitations, and attempts have been made to understand not only whether these features exist but also how they are arranged in space and positioned [5]. Rather than relying on individual value activations, CapsNets use small capsule structures to encode part–whole relationships and transform them. Hence, CapsNets can retain geometric information even with high amounts of contamination or topological transformations, which is a crucial and necessary supplementary advantage for any clinical application involving topological precision, such as lesion identification, organ segmentation, and tissue classification [5,6]. Despite this, the CapsNet architecture has yet to gain widespread clinical implementation. Their relatively high computational cost and difficulty in understanding their representations are still limiting factors for their implementation in clinical applications. Although previous studies have demonstrated improved performance compared to CNN architectures in dealing with spatial transformations, more efficient models and enhanced understandability are necessary for both architectures to play a role in clinical decision-making. CNNs and CapsNets play significant roles in boosting research on disease detection, segmentation and computer-aided diagnosis. Therefore, a formal comparison between these architectures is necessary to highlight their strengths and weaknesses. Ultimately, this study aims to highlight how these architectures can be combined or modified to develop more comprehensible AI models.

### 1.1. Background and Motivation

The interaction between artificial intelligence and medicine has evolved through several technological phases. The first phase was based on rule-based expert systems to obtain early computer-aided diagnosis systems, where the medical and clinical knowledge is encoded manually, and it necessitates an extensive number of experts in the field to have a large dataset that covers all cases concerning the area of study. Classical machine learning approaches, such as Support Vector Machines (SVMs) and Random Forests (RFs), rely on handcrafted imaging features to extract meaningful information that will help in diagnosis and medical support. The emergence of deep learning (DL) has produced a radical transformation in basic concepts, allowing neural networks (initially CNNs and, more recently, CapsNets) to learn hierarchical representations and features from medical images, which significantly improve the diagnostic performance and automated detection. Extensive research is ongoing in building technical capabilities to develop even more advanced applications in the medical domain, as it fundamentally addresses one of the most essential human needs: physical health. Artificial intelligence is a new and fast-growing field that comes with advanced capabilities to handle complex tasks at a level where computer-based performance surpasses that of even the most skilled humans. CNNs are a prevalent category of AI-based technology that are extremely efficient at processing visual input to perform many object recognition and image classification tasks, given their ability to stably learn underlying patterns in massive volumes of complex visual data. CapsNets are a more complex type of AI-based technology that improves some of CNNs’ shortcomings, most notably improving the ability to handle multi-scale features present in visual patterns. This makes CapsNets particularly suited for complex image recognition, including ambiguities [7,8], distortions [9], occlusions or low resolutions [10,11].

### 1.2. Structure of the Review

This paper aims to provide medical professionals and researchers with a comprehensive and systematic review of the development and current capabilities of AI-based technologies relying on CNNs and CapsNets. It focuses on their use in medical decision-making and how these models contribute to improving healthcare.

The remainder of this paper is structured as follows:

- **Section 2: Review Methodology**
  This section outlines the main approach followed to collect, select, and analyze the related studies for this review.
- **Section 3: Theoretical Background**
  Provides an overview of CNN and CapsNets architectures and explains how CapsNets address some of the major limitations of CNNs.
- **Section 4: Applications of CNNs and CapsNets in Medical Imaging**
  In this section, an introduction to the general topic of medical imaging and applications involving CNN and CapsNet would be discussed.
- **Section 5: Comparative Analysis of CNNs and CapsNets in Medical Image Analysis**
  This section will compare and examine the performance of CNNs and CapsNets regarding accuracy, precision, computational efficiency, and scalability. At the end we discuss the main advantages and shortcomings of both methods.
- **Section 6: Real-World Applications and Comparative Results**
  This aspect of the literature review will emphasize the implementations and case studies that show the comparative results and outputs of the systems that use CNN and CapsNets.
- **Section 7: Discussion and Future Perspectives**
  In order to address future outlooks, one section will be allocated to recapitulate the findings, point out new research patterns, and discuss potential future enhancements regarding the use of AI for the support of medical decisions.
- **Section 8: Conclusions**
  In order to conclude our review, major comparative insights and their potential impacts on future clinical and research developments are summarized in the following section.

**Main contributions of this review:**

- We provide a structured and pedagogical comparison between CNNs and CapsNets, describing their mathematical foundations, architectural principles, and operational differences.
- We synthesize recent medical imaging applications of both architectures across major diagnostic tasks, including classification, detection, and segmentation.
- We analyze the technical and clinical barriers that currently prevent CapsNets from achieving real-world deployment, highlighting computational constraints, lack of regulatory validation, and integration challenges in clinical workflows.
- We discuss emerging hybrid paradigms that combine CNNs, CapsNets, and attention-based models, and outline future research directions toward explainable, efficient, and clinically reliable AI decision-support systems.

## 2. Review Methodology

This review uses a structured approach to make it more rigorous and consistent, while still retaining its analytical and interpretable parts. The main purpose of this review is to analyze the development, progression, and evolution of two different variants of neural networks CNNs and CapsNets, concerning their usage within medical image support applications.

### 2.1. Scope and Temporal Coverage

The comprehensive review of the literature encompasses an array of studies conducted between the years 2018 and 2025. This era can be characterized by the significant advancement of CapsNets architectures and the subsequent integration of deep Convolutional Neural Networks within the medical domain. It is a period of recognition of the inadequacies associated with CNNs and the need for advanced spatial representation mechanisms.

### 2.2. Sources and Search Strategy

**Figure 1:**
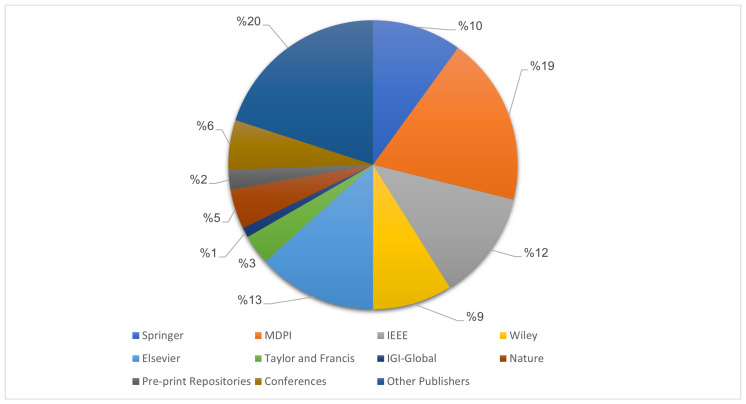
Publishers of the reviewed articles: percentage of their publications included in this review.

We used several databases for the selection of publications for this review, including IEEE Xplore, MDPI, ScienceDirect (Elsevier), SpringerLink, Wiley, IGI-Global, Taylor and Francis and Nature as well as preprint repositories like ResearchSquare, medRxiv, and ArXiv, to cover sources related to medical decision-making. Conference papers were also considered, and the search engines Semantic Scholar, Google Scholar and Academia were used to broaden the search. Figure 1 presents the percentage of publications from various publishers and preprint repositories analyzed in this study.

The search methodology employed Boolean operators alongside domain-specific terminology, incorporating keywords such as **CNN**, **Capsule Networks**, **Deep Learning**, **Medical Imaging**, **Segmentation**, **Classification**, **Decision making**, and **Clinical Diagnosis**. Subsequent to the initial search phase, publications were scrutinized for pertinence based on criteria in their titles, abstracts, and full texts. The focus was exclusively on studies pertaining to CNN or CapsNets architectures within the realm of medical imaging.

### 2.3. Inclusion and Exclusion Criteria

The criteria for choosing studies were based on relevance and scientific quality. The following inclusion criteria were applied:

- Published in English between 2018 and 2025;
- Related to the application or comparison of CNNs and CapsNets in medical imaging tasks such as classification, segmentation, or disease diagnosis;
- Systematic reviews specifically addressing CNN or CapsNets approaches;
- Reported measurable performance metrics (e.g., accuracy, sensitivity, specificity, precision, or F1-score);
- Used benchmark or publicly available datasets (e.g., BraTS, INbreast, ISIC);
- Contributed to research on interpretability, robustness, or hybrid deep learning architectures.

Studies were excluded if they:

- Were unrelated to biomedical imaging;
- Were purely theoretical without experimental validation;
- Were editorials, brief communications, or not peer-reviewed.

### 2.4. Analytical Approach

The comparative analysis was conducted across three main dimensions:

1. **Architectural Analysis**: Examining the model organization, feature retrieval procedures, and spatial encoding strategies.
2. **Performance Assessment**: Evaluating performance metrics and computational efficiency as presented in the literature.
3. **Clinical Relevance**: Discussing model interpretability, robustness to morphological variability, and potential applicability in real-world decision-support systems.

Unlike statistical aggregation, this review employs a conceptual synthesis, drawing comparisons across multiple studies to highlight major progress, persistent challenges, and future research directions in CNN and CapsNets development. Since this is a literature-based review, ethical approval was not required. No patient data or experimental procedures were involved. All references are properly cited and attributed to their original authors.

## 3. Theoretical Background

In this section, we will provide the foundational principles of two deep learning architectures that are the main focus of this review paper, namely CNNs and CapsNets. The following subsections give the theoretical background of each architecture in sequence.

### 3.1. Fundamentals of CNNs

**Figure 2:**
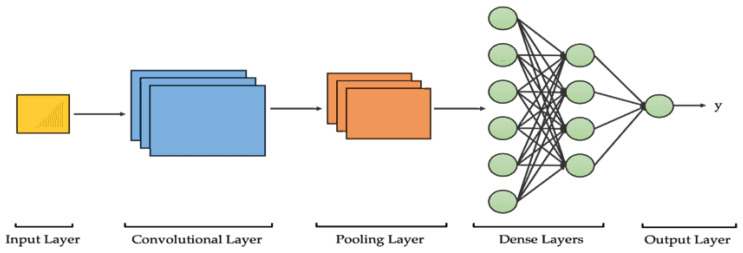
Schematic representation of the CNN hierarchical architecture. Convolutional and pooling layers extract low- to high-level features, which are then processed by fully connected layers for final classification.

CNNs are now considered the building block technology for contemporary vision systems. They grew out of biologically inspired models in the 1980s to mimic the hierarchical processing architecture of the human visual cortex. The notion that complex perceptions are generated through composition processes involving primitive patterns makes image understanding a natural task for a CNN. Following the pioneering efforts reported by LeCun et al., advances in data availability, computing resources, and training methodologies have collectively made CNNs the preeminent technology platform for image classification or pattern recognition. The capability to learn features automatically without human intervention has enabled impressive advances in a variety of applications including face or object recognition, natural scene understanding, or medical imaging [12,13,14,15]. A CNN can be thought of as a hierarchical model wherein each subsequent layer attempts to learn a new form of abstraction based on the input. Early layers usually focus on identifying local details in images like edges or corners. As we move to intermediate layers, these pieces of information are combined to convey textures or shapes. The final layers finally use these to represent larger concepts like organs or lesions [16,17]. These layers are generally followed by convolutional layers that are used to extract features, pooling layers to pool these features, and finally fully connected layers to map these features to output classes (Figure 2). Pooling operations play an essential role in CNNs by progressively reducing the spatial resolution of feature maps while preserving the most informative activations. Max pooling selects the highest activation within a local region, whereas average pooling computes the mean value. Both strategies reduce the number of parameters, improve computational efficiency, and introduce a degree of translational invariance, allowing the network to recognize relevant structures even when their precise spatial position changes. This mechanism helps retain the most salient information while discarding redundant details, which is particularly beneficial in medical image analysis where anatomical structures may appear at slightly varying locations across patients. From a functionality point of view, the difference between traditional feed-forward networks and CNNs lies in two architectural principles: local connectivity and weight sharing. Rather than processing entire images with filters or kernels, convolutional layers use small filters to analyze small parts of images. Finally, pooling layers improve translation invariance properties possessed by CNNs compared to traditional networks. The networks use optimization algorithms to learn filters to focus on specific features or areas within images based on which features are most informative. The effectiveness of CNNs can be demonstrated using larger benchmark datasets like ImageNet Large-Scale Visual Recognition Challenge (ILSVRC). Architectures like AlexNet, VGG, GoogLeNet, or ResNet illustrated the benefit of increasing model depth using residual or dense connections. In medical imaging, these concepts have found applications in domain-related tasks. Apart from lesion detection, tumor segmentation, or classification tasks in medical images like MRI, CT, or mammography images [18,19,20], there are numerous applications of these architectures. Also, one important component was transfer learning. Pre-trained models based on large datasets like natural images could easily learn smaller medical datasets. In terms of concepts, CNNs can actually be viewed as feature extractors that are arranged in layers to transform spatially extracted input features into more abstract features. Every subsequent layer implements a transformation that discovers more complex dependencies in images. In this way, a multi-level representation of semantics can actually be formed. In fact, this explains the effectiveness of generalization associated with CNNs. They are efficient and easy to modify because there are currently available frameworks that enable researchers to adapt quickly to new image problems. Further methodological studies have examined the optimization behavior and feature-learning dynamics of CNN layers across diverse image domains [21,22,23,24,25,26,27]. Although successful, there are structural weaknesses in CNNs which serve as driving forces for other architectural choices. Inherent to these networks are the characteristics of pooling layers or scalar activation functions which generally ignore specific details in the spatial arrangements of features identified. Although helpful in terms of translation invariance, these networks ignore orientation, scale factor, or geometric characteristics which are particularly relevant in biomedical contexts [28,29,30]. In these situations, features that are similar in appearance but differ in geometric arrangement are considered to be equal. Recently, there has been an effort to mitigate these inadequacies using architectural improvements like residual pathways, attention mechanisms, or multi-scale fusion. These are still improvements within the same convolutional framework. The need to not only encode what features are there but how these features are spatially arranged has led to emerging architectures. Among these architectures are those based on Capsule Networks (CapsNets), which are a natural progression or an extension of CNNs. The CNN architecture typically includes convolutional and pooling layers that extract and condense features, followed by fully connected layers that associate the learned representations with output categories. Despite their strong representation capacity, CNNs exhibit several limitations when applied to medical imaging. Their reliance on spatially invariant pooling makes them sensitive to large geometric deformations, and their internal feature representations remain difficult to interpret for clinicians. CNNs are also known to be vulnerable to adversarial perturbations, where imperceptible image modifications can produce incorrect diagnoses, and they generally require large-scale annotated datasets to achieve reliable performance in clinical settings. Moreover, high-performance CNN models typically require GPUs or TPU-based training infrastructures, and their deployment in real-time clinical workflows remains dependent on specialized hardware acceleration.

### 3.2. Fundamentals of Capsule Networks

**Figure 3:**
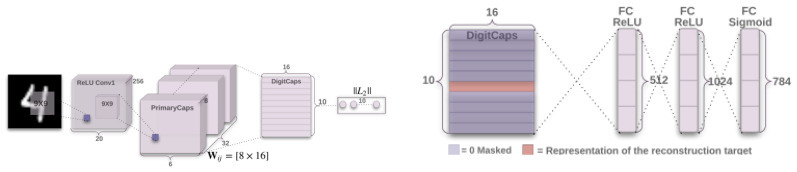
Illustrative structure of a Capsule Network. Capsules group neurons to encode both the presence and spatial configuration of features, while dynamic routing allows hierarchical information flow without losing spatial detail. Reprinted with permission from ref. [33]; Copyright 2017 NeurIPS.

CapsNets have brought about a paradigm shift in artificial neural networks (ANNs) and can be considered a remedy for several inherent flaws that prevail in conventional CNNs and fully connected networks (Figure 3). This novel piece of work was introduced by Geoffrey Hinton and his team for the first time in 2017. Thus, they have substantially improved CNNs. The basic architecture of CapsNets is designed to retain spatial hierarchical relationships. Although CNNs are known for their efficiency in feature extraction, their performance is largely dependent on pooling. This makes them vulnerable with respect to spatial information regarding object orientation. Thus, there is great relevance of CapsNets here, which can now solve these problems [31,32]. CapsNets can solve these problems by retaining information about the existence of features and spatial information. In addition to architectural differences, CNNs and Capsule Networks also rely on different loss functions during training. Conventional CNN classifiers are typically optimized using categorical cross-entropy, but this formulation assumes a balanced distribution of normal and pathological samples. Since medical datasets are frequently imbalanced, focal loss is commonly adopted to reduce the influence of easy negative examples and force the model to focus on clinically relevant minority cases. For segmentation problems such as tumor or lesion boundary detection, Dice loss is often preferred because it directly measures spatial overlap between prediction and ground truth, making it more appropriate for small or irregular anatomical structures. Capsule Networks, however, do not operate on scalar probabilities but on vector activations, and therefore cannot be trained with standard cross-entropy. Instead, they use the margin loss introduced by Sabour et al. [33] which enforces high vector lengths for the target class and suppresses all remaining capsules. This mechanism helps avoid overly confident predictions and improves robustness when detecting small abnormalities, a frequent difficulty in early cancer diagnosis. Some later studies have also explored spread loss as an alternative to margin loss in order to increase inter-class separation without relying on softmax. Beyond differences in the training objective, CapsNets also differ from CNNs in how they encode information: instead of scalar activations, they use vector-valued units called capsules. Each capsule is a small collection of neurons that represents a particular entity or object, with the magnitude of this vector representing the likelihood of its presence and the direction representing attributes such as orientation, angle, or size [33]. This allows CapsNets to identify objects irrespective of their orientation and capture the hierarchical relationships between a part and the whole. Hence, CapsNets can be considered to have improved representations compared with CNNs because they are more aligned with human visual processing. The key innovation in CapsNets is based on the dynamic routing by agreement algorithm developed by Sabour et al. (2017), which is used instead of the pooling operation in traditional CNNs. Here, lower-level capsule layers predict the vectors for higher-level capsule layers using transformation matrices, on which connections are strengthened if there is agreement with the prediction. Such communication between capsule layers makes it feasible for CapsNet models to have strong hierarchical representations without storing spatial information [34,35,36].

(1) $$c_{ij}=\frac{\exp(b_{ij})}{\sum_{k}\exp\left(b_{ik}\right)}$$

where $c_{ij}$ denotes the routing coefficient between capsule *i* and capsule *j*. The softmax ensures that the probabilities of routing from each lower-level capsule sum to one, strengthening consistent predictions and weakening inconsistent ones. Another critical role is played by the squashing non-linearity, which normalizes the vectors coming out of capsules such that short vectors tend towards zero, whereas long vectors tend towards a unit vector.

(2) $$v_{j}=\frac{∥s_{j}{∥}^{2}}{1+{∥s_{j}∥}^{2}}\frac{s_{j}}{∥s_{j}∥}$$

where $s_{j}$ is the total input to capsule *j*, and the squashing function keeps the output length within [0,1), preserving orientation while ensuring numerical stability.

### 3.3. Illustrative Example of Dynamic Routing

To provide an intuitive explanation of how dynamic routing operates, consider a simple case with two lower-level capsules $u_{1}$ and $u_{2}$, and two higher-level capsules $v_{1}$ and $v_{2}$. Each lower capsule predicts the output of each higher capsule through learned transformation matrices, producing four prediction vectors:

$${\hat{u}}_{1\to 1}=\left[0.9,\;0.1\right],\;{\hat{u}}_{1\to 2}=\left[0.2,\;0.8\right]$$

$${\hat{u}}_{2\to 1}=\left[0.8,\;0.3\right],\;{\hat{u}}_{2\to 2}=\left[0.1,\;0.9\right]$$

At the beginning of routing, all coupling coefficients are initialized uniformly:

$$c_{11}=c_{12}=c_{21}=c_{22}=0.5$$

The total input to each higher capsule is computed as:

$$s_{1}=0.5\cdot {\hat{u}}_{1\to 1}+0.5\cdot {\hat{u}}_{2\to 1}=\left[0.85,\;0.20\right]$$

$$s_{2}=0.5\cdot {\hat{u}}_{1\to 2}+0.5\cdot {\hat{u}}_{2\to 2}=\left[0.15,\;0.85\right]$$

After applying the squashing function (Equation (2)), the capsule activations become:

$$v_{1}=\left[0.78,\;0.18\right],\;v_{2}=\left[0.16,\;0.81\right]$$

The agreement between each prediction and output vector is given by the scalar product:

$$a_{1,1}=0.74,\;a_{1,2}=0.70,\;a_{2,1}=0.70,\;a_{2,2}=0.83$$

These agreements update the routing logits $b_{ij}$, which after softmax yield new coupling coefficients:

$$c_{1,1}=0.44,\;c_{1,2}=0.56,\;c_{2,1}=0.39,\;c_{2,2}=0.61$$

After only one routing iteration, both capsules begin to send more information to $v_{2}$, because it better agrees with their predicted votes. After two or three iterations, these coefficients converge, producing selective routing toward the capsule that best explains the input pattern. This simple numerical example illustrates how CapsNets achieve part-to-whole agreement without relying on max-pooling operations. Sabour et al. proposed the first applicable model of CapsNets in November 2017 with TensorFlow code, showing improved recognition performance on MNIST with affine transformations compared to traditional CNNs. They tested the robustness by introducing several variants with different **squashing functions** and by exploring alternative objective functions in subsequent capsule-based studies, including **spread loss**, which compares model-predicted class activations with the target activations and has been investigated as an alternative to the traditional softmax cross-entropy [37]. Their experiment verified the efficiency of CapsNet in parameter usage compared to CNNs for capturing geometric information.

(3) $$L_{k}=T_{k}\max(0,\;m^{+}-∥v_{k}{∥)}^{2}+\lambda \left(1-T_{k}\right)\max(0,\;∥v_{k}{∥-m^{-})}^{2}$$

where $T_{k}=1$ for the target class and 0 otherwise; $m^{+}$ and $m^{-}$ represent positive and negative margins, respectively. This margin-loss encourages correct capsules to have long output vectors and suppresses irrelevant activations. From an architectural perspective, a standard CapsNet model generally has three processing levels: The first processing level is a convolutional level. At this level, low-level features are extracted, similar to CNN models, but without any pooling operations. The second processing level is used to constitute the **PrimaryCaps** layer. It is mainly used to combine low-level features and obtain vector capsules that generally encode transformations and local patterns. The third processing level is called the **DigitCaps** or classification layer. At this processing level, high-level representations are attained via a process known as routing by agreement [33]. CapsNets can thus be regarded as a bridge combining CNNs and more understandable probabilistic models because they represent both the **what** (presence) and **where** (pose) information about features. Their inherent capacity to provide both rotational and translational invariance renders data augmentation and a feature pyramid unnecessary. Hence, CapsNets can represent small objects contained in larger entities, complex spatial transformations, and representations related to three-dimensional spaces compared to scalar architectures [34,35,36]. Although promising, CapsNets remain computationally expensive. Indeed, both the computations involved in routing and memory usage increase with the use of CapsNets, particularly when dealing with high-resolution images. Despite these limitations, several studies have proposed optimizations and efficient routing algorithms for CapsNets [32]. CapsNets are beneficial in medical imaging because they can preserve spatial information, allow perspective transformations, and model hierarchical part–whole relationships, which are critical for diagnostic imaging. CapsNets have demonstrated promising performance in locating small lesions, tumor identification across imaging modes, and enhancing the classification performance for geometric transformations in radiological images. They enable more robust and interpretable decision-support systems because CapsNets can maintain a more complex internal representation. With improvements in computational capabilities, CapsNets will inevitably play a significant role in model development for robust and interpretable clinical diagnosis systemss.

## 4. Applications of CNNs and CapsNets in Medical Imaging

This section reviews the most relevant applications of CNNs and Capsule Networks in medical imaging. Both architectures have contributed substantially to disease detection, image segmentation, and computer-aided diagnosis across diverse imaging modalities. The discussion highlights representative studies, datasets, and architectural adaptations that illustrate their respective strengths and limitations in real clinical contexts.

### 4.1. Disease Classification and Detection

Deep learning deployed in CNNs has shown potential in making more effective use of the large amounts of medical data that the healthcare sector can access. Different image modalities such as X-ray [38], CT [20,39], MRI [40], fundus [41], skin [42], endoscopy, dental [43,44], mammography [45], and pathology [46] images can be effectively processed and analyzed in deep learning-based CNN models, with further detailed classifications and detection of the diseases. Benchmark datasets such as BraTS for brain tumor MRI, ChestX-ray14 and COVIDx for thoracic disease detection, INbreast for mammographic cancer analysis, ISIC and HAM10000 for skin lesion classification, and CCE or HyperKvasir for endoscopic lesion detection have been widely used to validate CNN-based diagnostic models. According to the medical image types, models may perform slight adjustments to fit the need of experts, but the basic architecture of CNN models remains the same. One strength of CNNs is their ability to maintain high classification accuracy by using deep convolutional layers to extract image features directly from the raw pixels, which avoids time-consuming handcrafted feature extractions and filter design. In addition, deep learning models can leverage valuable transfer learning via fine-tuning and external datasets to resolve issues such as data insufficiency, hardware acceleration, and generalization when encountering relatively lower economic resources, small sample size, and finite computation power [47,48]. In view of the tremendous potential value and technical facility of CNNs, in this context, some early-stage trials, proofs of concept, and reviews proposed great possibilities and provided both intrinsic motivation and extrinsic knowledge of using deep learning models in medical decision-making as well as in disease prediction and classification. Nonetheless, despite the notable efforts by researchers and the real progress being made, reaching a mutual consensus on the potential value achieved by CNNs and other deep learning models for imaging diagnosis and improved patient treatment is still the subject of ongoing discussion. More importantly, the optimization, integration, and scalability of the CNNs, other types of deep learning models, and the practical decision-making process for healthcare professionals and their clients in the clinical setting may raise different requirements, standards, and needs compared with general IT applications, general level AI and ML implementations, and practice scenarios in other fields. CapsNets have emerged as a promising approach for disease detection and classification in medical imaging [49,50,51,52]. This new architecture proposes a novel means of feature representation and processing in medical images, which is highly beneficial and recommended for classifying and detecting various diseases. The key strength of this architecture is its ability to extract, capture, and maintain spatial information (the spatial arrangement of pixels), which is crucial for identifying and localizing abnormalities or disease in medical scans. This important spatial information is preserved through the unique capsule structure and the routing mechanism [53,54].

CapsNets architecture is well-suited for handling the challenges posed by medical imaging, such as variations in viewpoint, pose, and scale of anatomical structures. This is especially beneficial when dealing with diseases that manifest differently across various stages or in noisy image conditions. The capsules in CapsNets can encode the presence, pose, and spatial relationships of features, allowing for robust and accurate disease detection and classification. Moreover, CapsNets has demonstrated the ability to achieve high performance in disease detection tasks. Recent studies reported competitive results on datasets such as BraTS and Brain MRI (Kaggle) for tumor identification, 3D-IRCADb-01 for liver lesion classification, Messidor and ORIGA for retinal disease detection, and ISIC 2019 and HAM10000 for dermatological analysis. This efficiency is particularly valuable in medical applications where large, diverse datasets may be limited. CapsNet’s capacity to extract non-linear transformation features and capture discriminative characteristics makes it an attractive option for automated disease diagnosis systems.

Researchers are increasingly investigating CapsNets for diverse medical imaging applications, including the detection and classification of diseases across different modalities such as X-rays, MRI, and CT scans. The network’s natural capacity to capture complex spatial relationships and its resilience to image variations make it a promising tool for enhancing the accuracy and reliability of computer-aided diagnosis in healthcare.

With ongoing research, CapsNets show strong promise for improving automated disease detection and classification, enabling greater precision and effective diagnostic tools in clinical practice.

### 4.2. Medical Image Segmentation

Image segmentation is a critical task in medical image analysis, involving the partitioning of an image into multiple segments or regions of interest. While traditional methods such as thresholding, region growing, and edge detection have been used historically, the advent of deep learning, particularly CNNs, has revolutionized this field.

CNNs have emerged as powerful tools for image segmentation due to their ability to automatically learn hierarchical features from raw image data. Unlike traditional techniques that are based on handcrafted features, CNNs can capture complex spatial relationships and contextual information, making them particularly well-suited for the intricate nature of medical images.

Several CNN architectures have been developed specifically for medical image segmentation:

- **U-Net:** Introduced by Ronneberger et al. in 2015 [55,56], U-Net has become a cornerstone in medical image segmentation. Its symmetric encoder-decoder structure with skip connections allows for precise localization and effective use of context.
- **V-Net:** An extension of U-Net for 3D image segmentation [57], V-Net incorporates residual learning and is particularly useful for volumetric medical imaging data.
- **SegNet:** This architecture uses an encoder-decoder approach with indices-based unpooling [58], allowing for more efficient upsampling in the decoder.
- **DeepLab:** Employs atrous convolutions and atrous spatial pyramid pooling to capture multi-scale contextual information [59,60], beneficial for segmenting structures of varying sizes.

These CNN-based methods work by learning to classify each pixel (or voxel in 3D) into predefined classes, effectively creating a pixel-wise mask of the image. They have achieved excellent segmentation accuracy on benchmark datasets such as BraTS 2020 for brain tumors, DRIVE and ORIGA for retinal vessel and glaucoma segmentation, and ISIC for dermoscopic lesion boundaries. CNNs provide several advantages in the context of medical image segmentation:

- **Feature Extraction:** CNNs can learn and extract relevant features directly from the data in order to reduce the need for manual feature engineering.
- **Contextual awareness:** The hierarchical nature of CNNs allows them to capture both local and global context, crucial for accurate segmentation.
- **Adaptability:** CNNs can be fine-tuned for specific medical imaging modalities and anatomical structures.
- **End-to-end learning:** CNNs can be trained in an end-to-end manner, optimizing the entire segmentation pipeline simultaneously.

Nonetheless, challenges remain in applying CNNs to medical image segmentation:

- **Limited training data:** Medical imaging datasets are often small due to privacy concerns and the cost of annotation.
- **Class imbalance:** In many medical segmentation tasks, the region of interest may be much smaller than the background.
- **Three-dimensional data handling:** While 2D CNNs have shown success, efficiently processing 3D volumetric data remains challenging.

Despite the above-mentioned challenges, CNNs achieved remarkable success regarding various biomedical image segmentation tasks. For instance, CNNs were applied successfully for the segmentation of brain tumors from MRI images [61], lung nodules from CT images [62], and retinal artery–vein vessel segmentation from fundus images, supported by a high-quality public dataset (Fundus-AVSeg) [63].

As the research and development community progresses, there are expectations for improvement and development regarding the architecture and learning process of the CNNs, which could overcome the aforementioned challenges and allow the widespread use of such efficient tools for the analysis of biomedical images. The use of CapsNets for biomedical image segmentation leverages the accomplishments achieved by the latter for disease identification and diagnosis, and it also offers exclusive and innovative advantages for the segmentation of complex anatomical objects. Specific architectures for the segmentation tasks were developed for CapsNets, such as SegCaps [64], which adapted the original architecture of CapsNets for the use case concerning the problems of dense prediction. This model adapted the U-Net architecture, added skip connections, and replaced the classical convolutional layers with capsule layers for the conservation of the spatial information inside the network. In other works, the architecture named the **Capsule-UNet** [65] combines capsule layers with the U-Net architecture, and utilizes the advantages offered by this combination for the enhancement of segmentation accuracy. Experimental work has validated that the SegCaps achieved high Dice scores for the segmentation of brain tumors on the BraTS 2020 dataset, and the Capsule-UNet correctly segmented the retina and lung on the ORIGA and LUNA16 datasets, respectively. Multi-scale architectures for the segmentation using CapsNets have been designed, allowing the network to extract characteristics of the images for multiple resolutions, which is particularly effective for biomedical images concerning the objects of various sizes. This would allow for the generation of highly detailed segmentation maps, which are important for medical imaging concerning the boundary definition of tumors and for the definition of organ volumes. The ability to maintain spatial relations along with the hierarchy of the objects increased both the accuracy and detail of the segmentation maps, especially for the case of biomedical images. As research and development continue to progress, there are many prospects for improving and advancing the performance of CapsNets during image segmentation, especially in relation to increasing the efficiency for the processing of 3D biomedical images and strengthening simultaneous segmentation of multiple objects. Also, there are many prospects for the development of new and insightful capsule networks that would provide information concerning the decision-making processes by the network, which are important for the development and acceptance of the model by the biomedical community.

### 4.3. Multimodal Analysis and Computer-Aided Diagnosis

Medical Image Processing is at the core of early diagnosis of diseases and progress monitoring of pathologies. Recently, CNNs have significantly helped to develop this field over the last decade.

**Segmentation** is still one of the core tasks in a medical image analysis process. For example, architectures such as U-Net and a handful of variants all achieve excellent performance by accurately delineating areas of interest (tumors, organs, lesions, etc.) as they appear in the images. Their encoder–decoder structures enable the network not only to learn local patterns but also to capture more general contextual cues, leading to high accuracy and scalability in a variety of imaging paradigms [66,67,68,69,70].

**Classification** is also an important feature, where images are categorized in such a way as to reflect what is defined, for example, labeling the images as disease-present or -absent. CNNs are highly effective for this because they learn hierarchical features automatically from raw pixels. Some tried and tested algorithms, including AlexNet, VGGNet, and ResNet have demonstrated strong diagnosis performance on pneumonia, brain tumors, and diabetic retinopathy. They have been tested successfully on commonly performed datasets like ChestX-ray14, BraTS, and Messidor. Additionally, transfer learning improves performance even further; a transfer learning problem is performed where a network pre-trained on large natural-image corpora is fine-tuned to perform medical tasks in a single model. In addition to segmentation and sorting, object detection now relies on modern CNN-based techniques. Conventional methods (mostly based on handcrafted features and model-centric suggestions) also failed on accuracy and robustness. With the advent of detectors such as YOLO, Faster R-CNN, and SSD, among others, this terrain has been reshaped. And these models can automatically localize and classify the abnormalities at the same time by bounding the box (sometimes a rotated one), using region proposal networks or using anchor-based priors [71,72,73,74]. Their performance has been proven on standard datasets (e.g., ISIC for skin lesion localization, INbreast for mammography and BraTS for brain lesion detection). Nevertheless, the remaining challenges are the adaptation of natural image-training detectors to the special features of medical images as well as improvements in robustness to multi-scale and geometric variations. However, CNNs have undoubtedly advanced medical image analysis beyond previous research limitations [75] and could continue to be used further to overcome known limitations as well as improve diagnostic application. CapsNets have been proposed as a promising alternative for various medical imaging tasks in addition to CNNs. Its ability to model hierarchical–feature relationships is what makes them especially powerful (as well as relevant especially in the case of complex anatomical structures or fine pathological changes), where it is a key advantage. In real-life applications, CapsNet-based methods appear to have strong capabilities for different tasks. The methodologies have effectively been applied for use in disease recognition in endoscopic imaging (Kvasir-v2, CCE) analysis, ophthalmic imaging methods (Messidor, ORIGA) and brain lesion analysis with BraTS. Meanwhile, CapsNets have been studied for detecting anomalies within radiographs, allowing us to point out abnormal results that are not expected to meet a specific diagnostic standard. Recently, CapsNet architectures have been applied to multi-modal imaging such as MRI and PET scans for more complex diagnostic evaluations [50,76]. CapsNets have also been studied on how they can assist in image reconstruction in case of noise or lack of ability to obtain images, and that robust representation has brought advantages to this field [52,64]. An additional emerging trend is longitudinal work, and due to neural network sensitivity to spatial aspects, it provides a means to represent subtle structural changes over a period of time and therefore is quite useful for monitoring disease progression or response to treatments [77]. As the field further matures, CapsNets are also being applied to more complex analytical pipelines and are co-developed even with reinforcement learning or other AI-driven decision-making systems. The flexibility and development of CapsNets are underscored by these changes as the applications of these emerging technologies continue to broaden the field of automated medical image analysis.

## 5. Comparative Analysis of CNNs and CapsNets in Medical Image Analysis

Affine transformations such as rotation, scaling, and translation pose notable challenges in medical image analysis. Achieving robustness to these transformations is crucial for accurate diagnosis and treatment planning. In this section, we explore various modifications applied to CapsNets and CNNs to enhance their performance under affine transformations, and we also provide a conceptual comparison between these two deep learning architectures. Table 1 provides a structured conceptual overview of CNNs and CapsNets, summarizing their core architectural paradigms, computational characteristics, and generalization behavior. Table 2 then presents a more detailed comparative analysis of CNNs and CapsNets, highlighting key differences in the context of medical decision-making. Finally, Table 3 provides a comprehensive summary of the main modifications designed to improve robustness to affine transformations, detailing their intended impact and specific enhancements. Together, these tables offer a clear and structured roadmap for the quantitative and qualitative analyses that follow.

Representative benchmark datasets illustrating these behaviors, including BraTS, ISIC, INbreast, DRIVE, LUNA16, COVID-CT, and others, are detailed in the application-focused tables and subsections, ensuring traceability between architectures, data characteristics, and reported performance. However, reported improvements are not always consistent across datasets, imaging modalities, or evaluation metrics, indicating that performance differences between CNNs and CapsNets remain context-dependent rather than universally conclusive.

### 5.1. Performance Metrics

There are two common approaches to compare and contrast the performance of different machine learning models. One way is comparing them without considering the thresholds and aiming to obtain a figure that may be interpreted as a comprehensive performance measure. The other way is computing several performance scores by adopting different thresholds and imbalance considerations before evaluating the models. The latter approach is beneficial to healthcare practitioners as they can select and compare the model in question using the suitable trade-off point for the specific patient population, which will be explained later in this section. We present both methods and demonstrate how to compare models by visualizing The precision-recall curve (PRC) and its area under the curve (AUC), along with the receiver operating characteristic (ROC) curve and its corresponding AUC, Youden’s J statistic, and the scatter plot of the standardized (unrestricted) means of specificity, accuracy, sensitivity, positive predictive value (PPV), and negative predictive value (NPV).

AUC obtained from ROC (AUCROC) is the most established and well-known performance metric where it describes the probability of a model accurately classifying the samples (e.g., benign and malignant). In clinical practices, a model with an AUCROC larger than 0.7 is considered to have clinical utility or usefulness. Despite this, it is understandable that samplings are from an imbalanced class and that different patients are sensitive to the severity of a misclassification. For example, a disease prediction model should have much more robust performance for individuals with a high severe stage (i.e., needing high penalty for the advanced severity of the disease) compared to those at the early stage (i.e., lower penalty for the advanced stage). Hence, obtaining models that balance sample misclassification and developing models that achieve balanced performance across different severity levels should be taken into account.

### 5.2. Accuracy, Precision, Computational Efficiency, and Scalability

When comparing CNNs and CapsNets for medical image analysis, it is essential to examine their performance across several critical metrics: accuracy, precision, computational efficiency, and scalability.

**Accuracy and Precision:** CNNs have demonstrated high accuracy and precision in a range of medical imaging tasks, benefiting from extensive research and optimization. These networks excel in detecting and classifying features from medical images, particularly when large datasets are available. In contrast, CapsNets offer improved accuracy and precision in scenarios where spatial relationships and geometric transformations are critical. By maintaining hierarchical feature representations, CapsNets can achieve more nuanced interpretations, enhancing performance in complex image analysis tasks.

**Computational Efficiency:** CNNs are generally optimized and adjusted for performance and benefit from well-established hardware and software support. They are efficient in terms of training and inference due to mature optimization techniques. CapsNets, however, are computationally more intensive because of their dynamic routing algorithms and complex feature representations. This increased computational cost can impact training time and resource usage but may offer benefits in terms of robustness and feature extraction capabilities.

**Scalability:** CNNs are highly scalable, allowing them to effectively handle large datasets and a wide range of medical imaging applications. They can effectively handle increasing volumes of data with established architectures and methods. CapsNets show potential for scalability, but their computational complexity poses challenges. As dataset sizes and model complexity increase, managing the computational demands of CapsNets becomes more challenging, though ongoing advancements may mitigate these issues. In summary, while CNNs offer robust performance and efficiency, CapsNets provide advantages in feature preservation and spatial awareness. Both architectures have their strengths and weaknesses, and the choice between them should be guided by specific application requirements and resource constraints.

### 5.3. Strengths and Weaknesses of CNNs and CapsNets

CNNs utilize weight sharing and have a limited receptive field, which reduces the number of parameters and enhances efficiency. Yet, their sensitivity to translation and lack of spatial awareness can hinder their performance in tasks requiring an understanding of positional and spatial relationships. CapsNets address these limitations by enabling hierarchical feature learning, which improves position translation and deformation invariance. The dynamic routing mechanism in CapsNets strengthens feature learning, measures node activation levels, and provides more robust features. CNNs benefit from multi-resolution fusion methods, enhancing accuracy and reducing gradient loss by incorporating super-pixel-based segmentation methods into the shallow layers. CapsNets, alongside transfer learning models, help overcome the limitations of small datasets and improve robustness and accuracy through techniques like data enhancement and shuffled pooling. Fusion frameworks based on multiple deep model networks further enhance performance and applicability in various medical contexts. CapsNets address these limitations by enabling hierarchical feature learning, which improves position translation and deformation invariance. The dynamic routing mechanism in CapsNets strengthens feature learning, measures node activation levels, and provides more robust features.

## 6. Real-World Applications and Comparative Results

In this section, we provide a comprehensive comparative analysis of CNNs and CapsNets using real-world case studies in medical image analysis. We review a range of studies, detailing their application domain, architectures, datasets, and performance metrics. This comparative analysis aims to elucidate the strengths and limitations of each approach across various medical imaging tasks, providing insights into their effectiveness and applicability in real-world scenarios.

**Figure 4:**
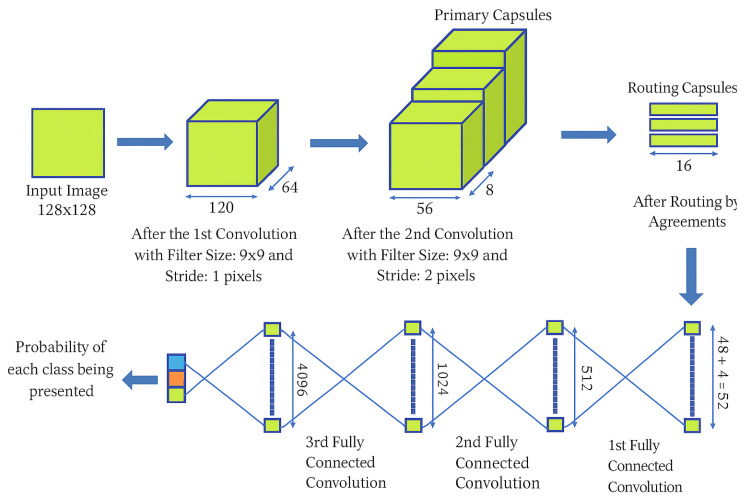
Proposed approach for brain tumor classification from MR images. Reprinted with permission from ref. [8]; Copyright 2020 Wiley.

The proposed CapsNet architecture by Goceri [8] addresses the challenge of classifying brain tumors, such as pituitary, glioma, and meningioma, from magnetic resonance images. This architecture incorporates three fully connected layers and employs an expectation-maximization-based dynamic routing algorithm, optimized with the Sobolev gradient-based SASGradD method. Using a dataset of 120 T1-weighted contrast-enhanced brain MR images, the study achieved an accuracy of 92.65%. However, manual segmentation of tumor regions was identified as a limitation due to its time-consuming and subjective nature. Further refinement of the CapsNet architecture and testing with larger datasets are suggested for improved outcomes (see Figure 4).

In another study, Aziz et al. [16] focused on accurately segmenting glioma tumors in MR images using a novel CapsNet architecture called SegCaps, which requires fewer training images compared to traditional CNNs like U-Net. Utilizing the BraTS2020 dataset, consisting of MRI scans from 369 patients, SegCaps achieved a Dice Similarity Coefficient (DSC) of 87.96% with only 1.5 million parameters, outperforming U-Net’s 85.56% DSC with 31 million parameters. Despite its slower routing algorithms and higher computational complexity, further optimization of SegCaps is recommended for complex medical imaging tasks.

Akinyelu and Bah [17] proposed CapsNetCovid, a deep learning model for diagnosing COVID-19 using X-ray and CT images. The architecture, comprising Conv layers, primary capsule layers, and a capsule layer digit, achieved high scores of 99.929% accuracy for CT images and 94.721% for X-ray images. Despite the model’s high performance, its decreased accuracy on augmented datasets highlights the need for further research to enhance generalization and robustness, particularly for multi-class classification.

Reis and Turk [78] introduced COVID-DSNet, a novel DCNN for rapid COVID-19 diagnosis using medical imaging techniques. Employing depthwise separable convolution and residual networks, the architecture achieved high accuracy (97.60%) in multi-class classification using CT, chest X-ray, and hybrid CT + CXR images. The study suggests future work involving data augmentation and transfer learning to improve model performance due to limitations such as small datasets and potential image noise.

**Figure 5:**
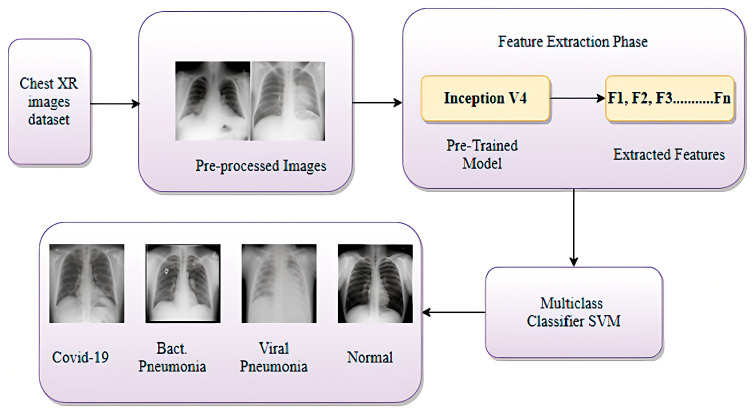
Proposed InceptionV4-SVM architecture for COVID-19 diagnosis from chest X-ray images. Reprinted with permission from ref. [79]; Copyright 2021 MDPI.

Kaur et al. [79] introduced C19D-Net, a deep learning model designed for diagnosing COVID-19 from chest X-ray images, as illustrated in Figure 5. Utilizing InceptionV4 for feature extraction and a multiclass SVM classifier, the model attained an accuracy of 96.24% across four classes: COVID-19, bacterial pneumonia, viral pneumonia, and normal. The study highlights the need for larger datasets to improve model performance and addresses potential observer variability in manual diagnoses.

Rahman et al. [18] addressed efficient breast cancer diagnosis from complex mammographic images using a CNN architecture, specifically ResNet-50. Utilizing the INbreast dataset, The study reported performance metrics including 93% accuracy, 93.86% specificity, and 93.83% sensitivity. Recommendations include exploring alternative networks like VGG and AlexNet for future improvements, while limitations involve potential performance variability across different datasets.

Swaraj et al. [80] focused on liver cancer classification from CT images using a CapsNet. The architecture, consisting of 41 layers, achieved over 86% classification accuracy using the 3D-IRCADb-01 dataset. The study suggests using false positive filters or training on larger datasets to mitigate the presence of false positives.

**Figure 6:**
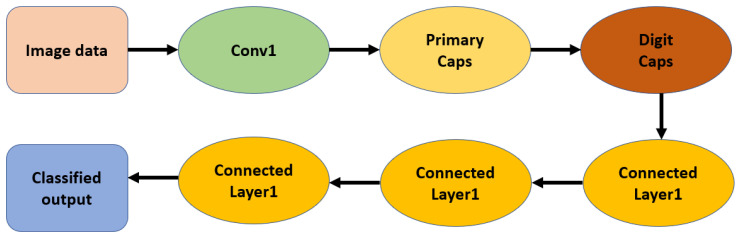
Structure diagram of CapsNet for liver cancer recognition. Reprinted with permission from ref. [81], Copyright 2022 MDPI.

Wang et al. [81] presented a liver cancer recognition method based on CapsNet as shown in Figure 6, achieving 92.9% accuracy for liver CT images compared to 87.6% for CNN. Despite potential overfitting, further validation on larger datasets is recommended to enhance generalization.

Iyyanar et al. [82] introduced a hybrid approach for effective glaucoma segmentation and classification using UNet++ and CapsNet. Utilizing the ORIGA dataset, the study achieved an overall accuracy of 97.6%. Future work should integrate Generative Adversarial Networks to enhance dataset availability and applicability to other datasets, addressing challenges posed by variations in image quality.

**Figure 7:**
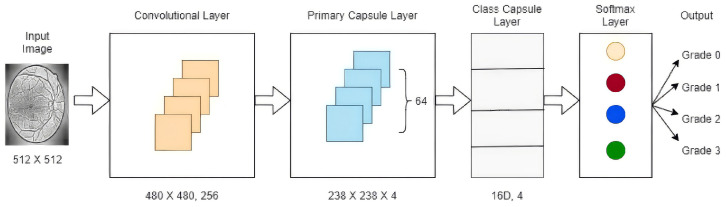
Modified CapsNet architecture for diabetic retinopathy classification. Reprinted with permission from ref. [83]; Copyright 2023 Springer Nature.

Kalyani et al. [83] focused on diabetic retinopathy detection and classification using a reformed capsule network architecture. The proposed architecture, avoiding pooling layers, achieved 97.98% accuracy for healthy retina using the Messidor dataset. Further training on additional datasets is recommended to improve classification across more stages of diabetic retinopathy (see Figure 7).

Mascarenhas et al. [84] introduced a CNN model for detecting colonic mucosal lesions and the presence of blood in colon capsule endoscopy (CCE) images. The study employed the Xception model, achieving a sensitivity of 96.3% and a specificity of 98.2% for mucosal lesion detection. Larger multicenter studies are recommended to validate findings and enhance CNN applications in clinical practice for CCE.

Afriyie et al. [85] proposed a denoising capsule network (Dn-CapsNets) for gastrointestinal tract disease recognition, achieving 94.16% accuracy using the Kvasir-v2 dataset. Further improvements on larger and more complex datasets like HyperKvasir are recommended, acknowledging the limitation of potential class imbalance.

Saraiva et al. [19] introduced a CNN model for the automatic identification and differentiation of small bowel lesions with distinct hemorrhagic potential using capsule endoscopy (CE) images. Utilizing the Xception model, the study achieved 99% accuracy. Larger studies are recommended to assess clinical impact and enhance generalizability across different CE systems. Mascarenhas et al. [84] presented another CNN model for the automatic detection of colonic mucosal lesions and blood in CCE images, achieving similar performance metrics. Prospective studies are recommended to confirm clinical applicability and enhance model robustness. Another method proposed by Hasnain et al. [86] in which a deep learning-based method for dental disease classification using X-ray images, achieving 97.87% accuracy. Further research to enhance model performance and generalizability is recommended due to the small dataset size.

Haghanifar [14] Introduced in this study is PaXNet, a groundbreaking and technologically advanced automated decision support system specifically designed for the purpose of dental caries detection in panoramic radiography. With an impressive accuracy rate of 86.05%, the findings of this study strongly advocate for the expansion of the dataset utilized to further enhance the capabilities and accuracy of PaXNet. Furthermore, it is recommended that segmentation methods be improved, resulting in even higher levels of precision and reliability. By following these proposed enhancements, the potential impact of PaXNet in the field of dental caries detection can be further amplified, ultimately leading to more effective diagnoses and improved patient outcomes.

AlSayyed et al. [87] employed an ensemble of CNN models to classify dental caries using oral photographs, achieving 97% accuracy. Larger, higher-quality datasets are recommended for improved model robustness and extending the framework to other medical domains.

Lan et al. [12] proposed FixCaps, an improved capsNet for skin cancer diagnosis, achieving 96.49% accuracy using the HAM10000 dataset. Further exploration of FixCaps’ generalization performance is recommended due to the limitation in its current evaluation scope.

Albraikan et al. [13] presented an Automated Deep Learning-Based Melanoma Detection and Classification (ADL-MDC) model, achieving 98.27% accuracy using the ISIC dataset. Further improvements in classification performance through advanced deep learning-based image segmentation techniques are suggested, addressing challenges in handling diverse image qualities.

Adla et al. [88] introduced a deep learning-based Computer-Aided Diagnosis (CAD) model for skin cancer detection, achieving 98.50% accuracy. Testing the model on larger datasets and in IoT environments is recommended, acknowledging challenges in segmenting lesions due to variations in texture, size, and color.

Alwakid et al. [15] proposed a deep learning-based method for detecting melanoma from dermoscopic images, achieving 0.86 accuracy for CNN using the dataset HAM10000. Further experiments on larger datasets are suggested, incorporating additional types of skin lesions to enhance model robustness.

Table 4 summarizes the real-world applications published in the literature.

CapsNets have shown considerable promise in medical imaging, attaining outstanding accuracy and efficiency across multiple applications. For instance, CapsNet models have reached up to 99.929% accuracy in diagnosing COVID-19 and 98.27% in melanoma detection. This high accuracy underscores the value of CapsNet in medical diagnostics. CapsNets are particularly advantageous due to their ability to capture spatial hierarchies and relationships between features. This capability is crucial in medical imaging tasks where understanding spatial context significantly impacts diagnostic precision.

Despite their strengths, CapsNets typically require longer training times and more computational resources compared to CNNs. Although this increased computational cost can be a drawback, the performance gains achieved by CapsNets often justify the additional resources. The improvements in accuracy and feature handling can be substantial, particularly in complex applications, making the investment in computational resources worthwhile. CNNs, while generally more efficient in terms of training time and computational resources, excel in tasks such as breast cancer detection and skin lesion classification. They deliver high accuracy with shorter training periods, which is advantageous in settings with limited computational power. However, CNNs can struggle with spatial variability and occlusions due to their less effective handling of hierarchical feature representations compared to CapsNets. In conclusion, while CNNs remain a powerful tool for many medical image analysis tasks due to their efficiency, CapsNets offer compelling advantages in applications requiring intricate spatial modeling and superior accuracy. Their ability to handle complex feature interactions and spatial hierarchies positions them as a promising alternative to traditional CNN methods. Future research should focus on optimizing CapsNet architectures to reduce training times and computational costs, while exploring integrations with advanced techniques like Generative Adversarial Networks (GANs) to enhance performance and applicability in medical imaging diagnostics.

## 7. Discussion and Future Perspectives

Overall, this review highlights the limitations of conventional CNNs in computer-aided diagnosis (CAD), particularly their dependence on large annotated datasets and their sensitivity to geometric transformations. In contrast, Capsule Networks preserve hierarchical part whole relationships and spatial orientation, improving robustness to viewpoint changes and occlusions. These properties make CapsNets theoretically attractive for medical imaging tasks that require precise characterization of shape, size, and spatial localization of abnormalities. Nevertheless, both architectures still face major research and deployment challenges, and no single approach can currently be considered universally optimal. Building on the findings summarized throughout this work, several emerging trends can be identified. Recent studies increasingly explore hybrid paradigms, such as CNN Transformer pipelines that combine convolutional filters with self-attention, or CapsNet Attention models that enhance routing mechanisms through adaptive relevance weighting. These developments indicate a convergence of deep learning paradigms rather than a strict paradigm shift. However, practical limitations persist: CapsNets remain computationally expensive, difficult to scale to large 3D volumes, and have not yet demonstrated consistent benefits across large multi-centre datasets. In clinical practice, deep learning deployment is still dominated by CNN-based solutions. Several systems including Transpara™ [89] for mammography analysis and Aidoc™ [90] for CT triage have been cleared by the U.S. Food and Drug Administration (FDA), the federal regulatory agency responsible for authorizing the clinical use of medical AI systems, and are already integrated into Picture Archiving and Communication Systems (PACS) in hospitals worldwide. No CapsNet-based diagnostic tool has yet achieved regulatory approval, mainly due to the lack of large-scale prospective validation, the computational cost of dynamic routing, and the absence of mature industrial software support. As a result, CapsNets remain confined to experimental research environments. Despite their theoretical promise, current CapsNet architectures face multiple barriers to real-world clinical deployment. Their iterative routing mechanisms significantly increase memory usage and inference latency, preventing real-time deployment on standard hospital hardware. In addition, no industrial-grade toolchains, pretrained model repositories, or regulatory submissions currently exist for Capsule Networks, and no prospective multi-centre clinical trials have validated their safety or generalizability. For these reasons, CapsNet-based systems cannot yet be integrated into clinical decision-support workflows or PACS infrastructures. Finally, in addition to performance considerations, future medical AI systems must address key ethical requirements, including transparency, robustness, accountability, and protection of patient privacy. Future research should therefore prioritize explainable and computationally efficient architectures that can be reliably deployed in heterogeneous clinical settings, supported by standardized evaluation protocols and regulatory frameworks.

### Emerging Hybrid Architectures and Future Directions

In more recent advances, deep learning has seen more focus on hybrid models, which attempt to harness the best of various paradigms. Taking the example of hybrid models involving the Transformer and CNN, models such as the CNN Transformer aim to harness the best of the feature-extraction capabilities of convolutions and the long-range dependencies modeled by the Transformer, which is particularly effective for applications such as tumor segmentation and histopathological image classification. Similarly, CapsNet Attention models aim to optimize the routing procedure and improve understandability by dynamically routing towards important areas. In doing so, models are able to correctly address noted limitations, including the limited receptive field of CNNs and the computational complexity of standard CapsNets, by promoting the utilization of feature selection and transforming.

Current studies indicate that such models may result not only in improvements to accuracy and efficiency but could potentially also support explainability and trustworthiness in clinical applications. As improvements and challenges simultaneously evolve, several future areas and priorities could potentially emerge. These areas and priorities include improving explainability and transparency as well as reliability and reproducibility under various conditions. Addressing bias and fairness of training data is also an important consideration. Other areas also include improving adaptability to distribution changes, assessing robustness and performing validation under various clinical conditions, and finally, improving patient safety and data confidentiality. In addition, integrations incorporating clinical prior knowledge, experience, and reasoning into models should also count towards future priorities. Additional multimodal patient inputs and hybrid models involving symbolic and data-driven models should also count towards future priorities. Lastly, guidelines and caution protocols should also count towards future priorities and could also act to support replicable, safe, and ethically sound clinical applications.

In summary, CapsNets show particular promise for tasks in which spatial dependencies directly influence diagnostic confidence, especially in problems involving subtle structural variations or shape-dependent abnormalities. Their potential is most evident in early-stage lesion characterization, microstructure detection, and cases where orientation or pose variability affects conventional CNN performance. Nonetheless, their integration into clinical workflows remains constrained by a limited body of large-scale validations and by the absence of standardized, deployment-ready implementations. These factors currently represent the main obstacles to their routine clinical adoption.

## 8. Conclusions

Recent advancements in CNNs and CapsNets have resulted in substantial progress in the domain of medical image analysis, especially within intricate decision-making contexts. CNNs continue to be the prevailing framework due to their robust empirical efficacy and well-established deployment infrastructure; however, their reliance on large annotated datasets and limited robustness to geometric variations represent important constraints. CapsNets were introduced to address these issues by preserving hierarchical spatial relationships and incorporating dynamic routing mechanisms, although their robustness and scalability require extensive validation on real medical datasets. Rather than being competing paradigms, CNNs and CapsNets can be considered as complementary. CNNs offer highly efficient feature extraction and have already demonstrated clinical utility, whereas CapsNets provide richer representational capabilities that can improve interpretability and fine-grained lesion characterization. However, both model families face practical barriers. CNNs remain vulnerable to adversarial perturbations and lack transparency owing to their black-box nature, whereas CapsNets suffer from high computational costs, limited scalability, and prolonged training times. Consequently, their successful translation into clinical workflows depends on rigorous validation, interoperability with decision support systems, and compliance with regulatory requirements. Future studies should focus on hybrid architectures that combine convolutional feature extractors, capsule-based geometric reasoning, and attention mechanisms derived from transformer m odels. Such unified systems may offer improved generalization, interpretability, and reliability in precision medicine. Large-scale prospective studies and interdisciplinary collaborations are essential for transitioning from theoretical advances to real clinical deployment. In addition to technical and clinical considerations, the adoption of deep learning in healthcare must comply with ethical and legal requirements. Issues related to privacy protection, algorithmic bias, and explainability of automated decisions are now considered essential components of medical AI validation. Therefore, future research should ensure that model development, evaluation, and deployment follow transparent and accountable procedures that preserve patient rights, while maintaining clinical safety and regulatory compliance.

## Figures and Tables

**Table 1:** Conceptual comparison of CNNs and CapsNets in medical imaging.

Aspect	CNNs	CapsNets
Core Principle	Hierarchical feature extraction using convolution and pooling; spatial hierarchies captured implicitly via shared weights.	Groups neurons into capsules; explicitly encodes part whole relationships and spatial configuration.
Transformation Handling	Invariance mainly through pooling and extensive data augmentation.	Equivariance to pose and rotation via capsule vectors and routing-by-agreement.
Interpretability	Moderate; relies on post-hoc saliency/attribution methods.	Higher; capsule outputs encode presence and pose, supporting more structured explanations.
Computational Efficiency	Highly optimized; scalable and efficient on large datasets.	More expensive due to iterative routing and vector operations.
Small/Imbalanced Data	Tends to overfit; improved by transfer learning and regularization.	Often more robust on limited data owing to richer internal representations.
Generalization	Strong on large, well-curated datasets; less robust to unseen geometric changes.	Improved robustness to viewpoint and deformation through explicit spatial modeling.
Clinical Applicability	Widely adopted in CAD pipelines for detection and segmentation.	Promising but not yet routine; complexity and lack of mature toolchains hinder deployment.

**Table 2:** Comprehensive comparison of CNNs and CapsNets in medical image analysis.

Characteristic	CNNs	CapsNets
Architecture	Uses convolutional layers, pooling layers, and fully connected layers	Uses capsules (groups of neurons) and dynamic routing algorithms
Spatial Relationships	Limited in capturing spatial hierarchies	Excels at capturing spatial hierarchies and relationships
Feature Detection	Detects features through convolutional filters	Encodes both the activation and the spatial relationship of features
Performance	Generally higher performance due to mature development and optimization	Currently less optimized, often resulting in lower performance
Hardware and Software Efficiency	Efficient and widely supported	Computationally intensive, with limited hardware and software support
Explainability	Limited interpretability	Potential for improved interpretability due to structured representation
Robustness to Variations	Sensitive to small variations in spatial structures	More robust to variations, capturing pose and orientation information
Applications in Medical Decision-Making	Widely applied in disease detection, classification, and image segmentation	Emerging applications, with potential for improved outcomes in similar tasks
Training Complexity	Well-established training methods and optimization techniques	More complex training, requiring dynamic routing and advanced optimization
Adoption in Clinical Settings	Widely adopted and validated in numerous clinical studies	Still in early stages of adoption, with ongoing research to validate effectiveness
Example Models	AlexNet, VGGNet, ResNet, Inception	CapsNet, SegCaps MCTDCapsNet
Use Case Examples	Tumor detection, organ segmentation, disease classification	Pose estimation, robust disease detection, spatial relationship analysis
Scalability	Highly scalable, well-suited for large datasets	Scalability challenges due to computational complexity
Transfer Learning Capability	Excellent, with many pre-trained models available	Limited, as the field is still developing
Data Augmentation Requirements	Moderate to high	Potentially lower due to inherent rotational invariance
Handling of 3D Imaging	Requires specialized architectures (e.g., 3D CNNs)	Potentially more natural handling of 3D structures
Multimodal Integration	Well-established techniques available	Promising, but less explored
Uncertainty Quantification	Requires additional techniques (e.g., dropout)	Potentially more inherent through capsule activations
Few-shot Learning	Generally requires large datasets	Potentially better with limited data due to efficient parameter sharing
Computational Resource Requirements	Well-optimized, moderate requirements	Higher requirements, especially during training
Handling of Class Imbalance	Requires specific techniques (e.g., weighted loss)	May handle imbalance better due to equivariance properties
Interpretability Tools	Various tools available (e.g., Grad-CAM)	Emerging tools, potentially more intuitive due to capsule structure
Regulatory Compliance	Established frameworks for validation	Frameworks still developing
Longitudinal Analysis Capability	Requires specific architectures	Potentially better suited due to pose preservation

**Table 3:** Various CapsNet and CNN modifications to enhance affine transformation robustness.

Modification Type	Modification	Description	Impact on Robustness to Affine Transformations
**CapsNet** **modifications**	Dynamic routing with RBA	Routing-by-agreement (RBA) variation of dynamic routing.	Enhances capsule consensus and adaptability to variations in position, rotation, and scale.
	Aff-CapsNets	Affine CapsNets.	Reduces the number of parameters while increasing resistance to affine changes.
	Transformation-aware capsules	Capsules explicitly designed to handle affine transformations.	Detects and applies suitable transformations, boosting invariance to affine changes.
	Capsule-capsule transformation (CCT)	Adaptive transformation between capsules.	Effectively manages varying degrees of affine transformations.
	Margin loss regularization	Adds margin loss terms during training.	Promotes larger margins between capsules, enhancing resistance to affine changes.
	Capsule routing with EM routing	Utilizes an EM-like algorithm for capsule routing.	Strengthens the capsule agreement process and feature learning, improving robustness to affine transformations.
	Self-routing	A supervised, non-iterative routing method.	Eliminates the need for inter-capsule agreement, similar to mixture of experts (MoE), thereby enhancing robustness.
	Adversarial capsule networks	Combines CapsNet with adversarial training techniques.	Trains against adversarial affine transformations, enhancing feature robustness.
	Capsule dropouts	Applies dropouts to capsules during training.	Improves generalization and robustness by preventing capsule co-adaptations.
	Capsule reconstruction	Augments CapsNet with a reconstruction loss term.	Preserves spatial information, improving robustness to affine transformations.
	Capsule attention mechanism	Incorporates attention mechanisms into capsules.	Focuses on important features, aiding robustness against affine transformations.
**CNN** **modifications**	Spatial transformer networks (STN)	Introduces learnable modules to perform spatial transformations.	Enhances the ability to learn transformations, improving invariance to affine changes.
	Data augmentation	Applies random transformations (e.g., rotation, scaling) to training data.	Trains on diverse affine-transformed images, increasing robustness.
	Deformable convolutions	Modifies convolutional filters to adapt to spatial transformations.	Enhances feature extraction by allowing filters to deform, improving robustness to affine changes.
	Affine invariant layers	Uses layers specifically designed to be affine invariant.	Integrates affine invariance directly into the network, improving resilience to affine changes.
	Local binary patterns (LBP)	Extracts texture features that are robust to affine transformations.	Focuses on texture rather than spatial arrangement, increasing robustness.
	Rotation equivariant CNNs	Incorporates rotation-equivariant filters in the architecture.	Enhances handling of rotational transformations, improving robustness to affine changes.
	Adversarial training	Trains the network with adversarial examples, including affine transformations.	Learns robust features by resisting adversarial and affine transformations.
	Pooling techniques (e.g., max pooling)	Uses pooling layers to reduce spatial dimensions.	Provides partial invariance to translations and distortions, aiding robustness to affine changes.
	SIFT-like feature extraction	Utilizes SIFT-like (Scale-Invariant Feature Transform) methods.	Enhances invariance to scale and rotation, improving robustness to affine changes.
	Attention mechanisms	Incorporates attention layers to focus on important features.	Improves focus on relevant features, enhancing robustness to affine transformations.

**Table 4:** Summary of performance metrics for various CNNs and CapsNets.

Study	Application/Problem Definition	Architecture and Parameters	Dataset	Performance Metrics	Recommendations/Limitations
Goceri [8]	Brain tumor classification (pituitary, glioma, meningioma)	3 fully connected layers, dynamic routing, SASGradD method	120 T1-weighted contrast-enhanced brain MR images	Accuracy: 92.65%	Manual segmentation is time-consuming and subjective; further refinement and larger datasets needed
Aziz et al. [16]	Glioma tumor segmentation in MR images	SegCaps, fewer training images than U-Net	BraTS2020 dataset (MRI scans from 369 patients)	DSC: 87.96%, Parameters: 1.5 M	Slower routing algorithms, higher computational complexity; further optimization needed
Akinyelu & Bah [17]	COVID-19 detection based on CT and X-ray imaging	Conv layers, primary capsule layers, digit capsule layer	CT and X-ray images	Accuracy: 99.929% (CT), 94.721% (X-ray)	Decreased accuracy on augmented datasets; further research for generalization and robustness needed
Reis & Turk [78]	COVID-19 diagnosis using medical imaging	Depthwise separable convolution, residual networks	CT, chest X-ray, hybrid CT + CXR images	Accuracy: 97.60%	Small datasets, potential image noise; suggests data augmentation and transfer learning
Kaur et al. [79]	COVID-19 diagnosis from chest X-ray images	InceptionV4, multiclass SVM classifier	Chest X-ray images	Accuracy: 96.24% (four classes)	Needs larger datasets; potential observer variability in manual diagnoses
Rahman et al. [18]	Breast cancer diagnosis from mammographic images	ResNet-50	INbreast dataset	Accuracy: 93%, Specificity: 93.86%, Sensitivity: 93.83%	Potential performance variability across datasets; exploring alternative networks (VGG, AlexNet) recommended
Swaraj et al. [80]	Liver cancer classification from CT images	CapsNet, 41 layers	3D-IRCADb-01 dataset	Accuracy: 86%+	Suggests using false positive filters or larger datasets to mitigate false positives
Wang et al. [81]	Liver cancer recognition	CapsNet	Liver CT images	Accuracy: 92.9% (CapsNet), 87.6% (CNN)	Potential overfitting; further validation on larger datasets recommended
Iyyanar et al. [82]	Glaucoma segmentation and classification	UNet++, CapsNet	ORIGA dataset	Accuracy: 97.6%	Integrating Generative Adversarial Networks to enhance dataset availability and applicability
Kalyani et al. [83]	Diabetic retinopathy detection and classification	Reformed capsNet architecture, avoids pooling layers	Messidor dataset	Accuracy: 97.98% (healthy retina)	Further training on additional datasets recommended for more stages of diabetic retinopathy
Mascarenhas et al. [84]	Detection of colonic mucosal lesions and blood in CCE images	CNN model, Xception	CCE images	Sensitivity: 96.3%, Specificity: 98.2%	Larger multicenter studies recommended for validation and enhancing clinical applications
Afriyie et al. [85]	Gastrointestinal tract disease recognition	Dn-CapsNets	Kvasir-v2 dataset	Accuracy: 94.16%	Further improvements on larger and more complex datasets like HyperKvasir recommended
Saraiva et al. [19]	Identification and differentiation of small bowel lesions	CNN model, Xception	Capsule endoscopy (CE) images	Accuracy: 99%	Larger studies recommended to assess clinical impact and enhance generalizability
Mascarenhas et al. [84]	Detection of colonic mucosal lesions and blood in CCE images	CNN model	CCE images	Similar performance metrics to prior study	Prospective studies recommended to confirm clinical applicability and enhance model robustness
Hasnain et al. [86]	Dental disease classification	Deep learning-based approach	X-ray images	Accuracy: 97.87%	Small dataset size; further research to enhance performance and generalizability recommended
Haghanifar [14]	Dental caries detection in panoramic radiography	PaXNet	Panoramic radiography	Accuracy: 86.05%	Suggests expanding the dataset and improving segmentation methods for better accuracy
AlSayyed et al. [87]	Dental caries classification using oral photographs	CNN ensemble models	Oral photographs	Accuracy: 97%	Larger, higher-quality datasets recommended; suggests extending framework to other medical domains
Lan et al. [12]	Skin cancer diagnosis	FixCaps	HAM10000 dataset	Accuracy: 96.49%	Further exploration of generalization performance recommended due to limitation in current evaluation scope
Albraikan et al. [13]	Melanoma detection and classification	ADL-MDC model	ISIC dataset	Accuracy: 98.27%	Further improvements in classification performance through advanced deep learning-based image segmentation techniques suggested
Adla et al. [88]	Skin cancer detection	Deep learning-based CAD model	Skin images	Accuracy: 98.50%	Testing on larger datasets and in IoT environments recommended; challenges in segmenting lesions due to variations in texture, size, and color
Alwakid et al. [15]	Melanoma detection using dermoscopic images	Deep learning-based approach	HAM10000 dataset	Accuracy: 0.86 (CNN)	Further experiments on larger datasets suggested; incorporating additional types of skin lesions to enhance model robustness

## Data Availability

No new data were created or analyzed in this study. Data sharing is not applicable to this article.

## References

[1] Kumar Y., Koul A., Singla R., Ijaz M.F. (2023). Artificial intelligence in disease diagnosis: A systematic literature review, synthesizing framework and future research agenda. J. Ambient. Intell. Humaniz. Comput..

[2] Chan H.P., Hadjiiski L.M., Samala R.K. (2020). Computer-aided diagnosis in the era of deep learning. Med. Phys..

[3] Battineni G., Sagaro G.G., Chinatalapudi N., Amenta F. (2020). Applications of machine learning predictive models in the chronic disease diagnosis. J. Pers. Med..

[4] Zhou S.K., Greenspan H., Davatzikos C., Duncan J.S., Van Ginneken B., Madabhushi A., Summers R.M. (2021). A review of deep learning in medical imaging: Imaging traits, technology trends, case studies with progress highlights, and future promises. Proc. IEEE.

[5] Patrick M.K., Adekoya A.F., Mighty A.A., Edward B.Y. (2022). Capsule networks—A survey. J. King Saud-Univ.-Comput. Inf. Sci..

[6] Sun Z., Zhao G., Scherer R., Wei W., Woźniak M. (2022). Overview of capsule neural networks. J. Internet Technol..

[7] Mazzia V., Salvetti F., Chiaberge M. (2021). Efficient-capsnet: Capsule network with self-attention routing. Sci. Rep..

[8] Goceri E. (2020). CapsNet topology to classify tumours from brain images and comparative evaluation. IET IMage Process..

[9] Ali R., Manikandan A., Xu J. (2023). A novel framework of adaptive fuzzy-GLCM segmentation and fuzzy with capsules network (F-CapsNet) classification. Neural Comput. Appl..

[10] Yuan Y., Chu J., Leng L., Miao J., Kim B.G. (2020). A scale-adaptive object-tracking algorithm with occlusion detection. EURASIP J. Image Video Process..

[11] Zeng D., Veldhuis R., Spreeuwers L. (2021). A survey of face recognition techniques under occlusion. IET Biom..

[12] Lan Z., Cai S., He X., Wen X. (2022). FixCaps: An improved capsules network for diagnosis of skin cancer. IEEE Access.

[13] Albraikan A.A., Nemri N., Alkhonaini M.A., Hilal A.M., Yaseen I., Motwakel A. (2023). Automated deep learning based melanoma detection and classification using biomedical dermoscopic images. Comput. Mater. Contin..

[14] Haghanifar A. (2022). Automated Teeth Extraction and Dental Caries Detection in Panoramic X-Ray. Ph.D. Thesis.

[15] Alwakid G., Gouda W., Humayun M., Sama N.U. (2022). Melanoma detection using deep learning-based classifications. Healthcare.

[16] Aziz M.J., Zade A.A.T., Farnia P., Alimohamadi M., Makkiabadi B., Ahmadian A., Alirezaie J. (2021). Accurate automatic glioma segmentation in brain MRI images based on CapsNet. Proceedings of the 2021 43rd Annual International Conference of the IEEE Engineering in Medicine & Biology Society (EMBC).

[17] Akinyelu A.A., Bah B. (2023). COVID-19 diagnosis in computerized tomography (CT) and X-ray scans using capsule neural network. Diagnostics.

[18] Rahman H., Naik Bukht T.F., Ahmad R., Almadhor A., Javed A.R. (2023). Efficient breast cancer diagnosis from complex mammographic images using deep convolutional neural network. Comput. Intell. Neurosci..

[19] Saraiva M.J.M., Afonso J., Ribeiro T., Ferreira J., Cardoso H., Andrade A.P., Macedo G. (2021). Deep learning and capsule endoscopy: Automatic identification and differentiation of small bowel lesions with distinct haemorrhagic potential using a convolutional neural network. BMJ Open Gastroenterol..

[20] An Q., Chen W., Shao W. (2024). A deep convolutional neural network for pneumonia detection in x-ray images with attention ensemble. Diagnostics.

[21] Li Z., Liu F., Yang W., Peng S., Zhou J. (2021). A survey of convolutional neural networks: Analysis, applications, and prospects. IEEE Trans. Neural Netw. Learn. Syst..

[22] Yin H., Gong Y., Qiu G. (2020). Fast and efficient implementation of image filtering using a side window convolutional neural network. Signal Process..

[23] Romero D.W., Kuzina A., Bekkers E.J., Tomczak J.M., Hoogendoorn M. (2021). Ckconv: Continuous kernel convolution for sequential data. arXiv.

[24] Taye M.M. (2023). Theoretical understanding of convolutional neural network: Concepts, architectures, applications, future directions. Computation.

[25] Santra S., Hsieh J.W., Lin C.F. (2021). Gradient descent effects on differential neural architecture search: A survey. IEEE Access.

[26] Jagadeesan M., Razenshteyn I., Gunasekar S. Inductive bias of multi-channel linear convolutional networks with bounded weight norm. Proceedings of the Conference on Learning Theory.

[27] Debnath T., Reza M.M., Rahman A., Beheshti A., Band S.S., Alinejad-Rokny H. (2022). Four-layer ConvNet to facial emotion recognition with minimal epochs and the significance of data diversity. Sci. Rep..

[28] Jena B., Nayak G.K., Saxena S. (2022). Convolutional neural network and its pretrained models for image classification and object detection: A survey. Concurr. Comput. Pract. Exp..

[29] Zhao Q., Shang Z. (2021). Deep learning and its development. J. Phys. Conf. Ser..

[30] Akhtar N., Mian A., Kardan N., Shah M. (2021). Advances in adversarial attacks and defenses in computer vision: A survey. IEEE Access.

[31] Hinton G. (2023). How to represent part-whole hierarchies in a neural network. Neural Comput..

[32] Taher O., Özacar K. (2024). HeCapsNet: An enhanced capsule network for automated heel disease diagnosis using lateral foot X-Ray images. Int. J. Imaging Syst. Technol..

[33] Sabour S., Frosst N., Hinton G.E. Dynamic routing between capsules. Proceedings of the Advances in Neural Information Processing Systems.

[34] Shiri P. (2022). Optimizing Capsule Networks. Ph.D. Thesis.

[35] Sood K., Fiaidhi J. (2020). Capsule Networks: An Alternative Approach to Image Classification Using Convolutional Neural Networks. TechRxiv.

[36] Tran M., Vo-Ho V.K., Quinn K., Nguyen H., Luu K., Le N. (2024). CapsNet for medical image segmentation. Deep Learning for Medical Image Analysis.

[37] Hinton G.E., Sabour S., Frosst N. Matrix Capsules with EM Routing. Proceedings of the International Conference on Learning Representations (ICLR).

[38] Bhardwaj P., Kaur A. (2021). A novel and efficient deep learning approach for COVID-19 detection using X-ray imaging modality. Int. J. Imaging Syst. Technol..

[39] Hilmizen N., Bustamam A., Sarwinda D. (2020). The multimodal deep learning for diagnosing COVID-19 pneumonia from chest CT-scan and X-ray images. Proceedings of the 2020 3rd International Seminar on Research of Information Technology and Intelligent Systems (ISRITI).

[40] Huang J., Fang Y., Wu Y., Wu H., Gao Z., Li Y., Del Ser J., Xia J., Yang G. (2022). Swin transformer for fast MRI. Neurocomputing.

[41] Li T., Bo W., Hu C., Kang H., Liu H., Wang K., Fu H. (2021). Applications of deep learning in fundus images: A review. Med. Image Anal..

[42] Solnik M., Paduszyńska N., Czarnecka A.M., Synoradzki K.J., Yousef Y.A., Chorągiewicz T., Rejdak R., Toro M.D., Zweifel S., Dyndor K. (2022). Imaging of uveal melanoma—Current standard and methods in development. Cancers.

[43] Szabó V., Orhan K., Dobó-Nagy C., Veres D.S., Manulis D., Ezhov M., Sanders A., Szabó B.T. (2025). Deep Learning-Based Periapical Lesion Detection on Panoramic Radiographs. Diagnostics.

[44] Çetinkaya İ., Çatmabacak E.D., Öztürk E. (2025). Detection of Fractured Endodontic Instruments in Periapical Radiographs: A Comparative Study of YOLOv8 and Mask R-CNN. Diagnostics.

[45] Alwateer M., Bamaqa A., Farsi M., Aljohani M., Shehata M., Elhosseini M.A. (2025). Transformative Approaches in Breast Cancer Detection: Integrating Transformers into Computer-Aided Diagnosis for Histopathological Classification. Bioengineering.

[46] Abhisheka B., Biswas S.K., Purkayastha B., Das D., Escargueil A. (2024). Recent trend in medical imaging modalities and their applications in disease diagnosis: A review. Multimed. Tools Appl..

[47] Deng Y., Lu L., Aponte L., Angelidi A.M., Novak V., Karniadakis G.E., Mantzoros C.S. (2021). Deep transfer learning and data augmentation improve glucose levels prediction in type 2 diabetes patients. npj Digit. Med..

[48] Sufian A., Ghosh A., Sadiq A.S., Smarandache F. (2020). A survey on deep transfer learning to edge computing for mitigating the COVID-19 pandemic. J. Syst. Archit..

[49] Yadav S., Dhage S. (2024). TE-CapsNet: Time efficient capsule network for automatic disease classification from medical images. Multimed. Tools Appl..

[50] Saif A.F.M., Imtiaz T., Rifat S., Shahnaz C., Zhu W.P., Ahmad M.O. (2021). CapsCovNet: A modified capsule network to diagnose Covid-19 from multimodal medical imaging. IEEE Trans. Artif. Intell..

[51] Aksoy B., Salman O.K.M. (2021). Detection of COVID-19 Disease in Chest X-Ray Images with capsul networks: Application with cloud computing. J. Exp. Theor. Artif. Intell..

[52] Shah M., Bhavsar N., Patel K., Gautam K., Chauhan M. (2023). Modern Challenges and Limitations in Medical Science Using Capsule Networks: A Comprehensive Review. Proceedings of the International Conference on Image Processing and Capsule Networks.

[53] Wu Y., Cen L., Kan S., Xie Y. (2023). Multi-layer capsule network with joint dynamic routing for fire recognition. Image Vis. Comput..

[54] Chen R., Shen H., Zhao Z.Q., Yang Y., Zhang Z. (2024). Global routing between capsules. Pattern Recognit..

[55] Siddique N., Paheding S., Elkin C.P., Devabhaktuni V. (2021). U-net and its variants for medical image segmentation: A review of theory and applications. IEEE Access.

[56] Du X., Cheng K., Zhang J., Wang Y., Yang F., Zhou W., Lin Y. (2025). Infrared Small Target Detection Algorithm Based on Improved Dense Nested U-Net Network. Sensors.

[57] Mohammed K.K., Hassanien A.E., Afify H.M. (2021). A 3D image segmentation for lung cancer using V. Net architecture based deep convolutional networks. J. Med. Eng. Technol..

[58] He F., Wang W., Ren L., Zhao Y., Liu Z., Zhu Y. (2024). CA-SegNet: A channel-attention encoder—Decoder network for histopathological image segmentation. Biomed. Signal Process. Control.

[59] Li Y., Li P., Wang H., Gong X., Fang Z. (2025). CAML-PSPNet: A Medical Image Segmentation Network Based on Coordinate Attention and a Mixed Loss Function. Sensors.

[60] Yamada T., Yoshimura T., Ichikawa S., Sugimori H. (2025). Improving Cerebrovascular Imaging with Deep Learning: Semantic Segmentation for Time-of-Flight Magnetic Resonance Angiography Maximum Intensity Projection Image Enhancement. Appl. Sci..

[61] Ranjbarzadeh R., Caputo A., Tirkolaee E.B., Ghoushchi S.J., Bendechache M. (2023). Brain tumor segmentation of MRI images: A comprehensive review on the application of artificial intelligence tools. Comput. Biol. Med..

[62] Halder A., Chatterjee S., Dey D., Kole S., Munshi S. (2020). An adaptive morphology based segmentation technique for lung nodule detection in thoracic CT image. Comput. Methods Programs Biomed..

[63] Deng Z., Gao W., Gong Z., Gan R., Chen L., Zhang S., Ma L. (2025). A Fundus Image Dataset for AI-based Artery-Vein Vessel Segmentation. Sci. Data.

[64] Poiret C., Bouyeure A., Patil S., Boniteau C., Duchesnay E., Grigis A., Lemaitre F., Noulhiane M. (2024). Attention-gated 3D CapsNet for robust hippocampal segmentation. J. Med. Imaging.

[65] Avesta A., Hossain S., Lin M., Aboian M., Krumholz H.M., Aneja S. (2023). Comparing 3D, 2.5 D, and 2D approaches to brain image auto-segmentation. Bioengineering.

[66] Kourounis G., Elmahmudi A.A., Thomson B., Hunter J., Ugail H., Wilson C. (2023). Computer image analysis with artificial intelligence: A practical introduction to convolutional neural networks for medical professionals. Postgrad. Med. J..

[67] Olveres J., González G., Torres F., Moreno-Tagle J.C., Carbajal-Degante E., Valencia-Rodríguez A., Escalante-Ramírez B. (2021). What is new in computer vision and artificial intelligence in medical image analysis applications. Quant. Imaging Med. Surg..

[68] Hassan E., Shams M.Y., Hikal N.A., Elmougy S. (2022). A novel convolutional neural network model for malaria cell images classification. Comput. Mater. Contin..

[69] Kodipalli A., Fernandes S.L., Dasar S.K., Ismail T. (2023). Computational framework of inverted fuzzy C-means and quantum convolutional neural network towards accurate detection of ovarian tumors. Int. J. E-Health Med. Commun. (IJEHMC).

[70] Vadlamudi S.H., Sai Souhith Reddy Y., Ajith Sai Kumar Reddy P., Periasamy P., Vali Mohamad N.M. (2022). Automatic liver tumor segmentation and identification using fully connected convolutional neural network from CT images. Concurr. Comput. Pract. Exp..

[71] Babulal K.S., Das A.K. (2022). Deep learning-based object detection: An investigation. Proceedings of the Futuristic Trends in Networks and Computing Technologies: Select Proceedings of Fourth International Conference on FTNCT 2021.

[72] Kaur A., Singh Y., Neeru N., Kaur L., Singh A. (2022). A survey on deep learning approaches to medical images and a systematic look up into real-time object detection. Arch. Comput. Methods Eng..

[73] Tayal A., Gupta J., Solanki A., Bisht K., Nayyar A., Masud M. (2022). DL-CNN-based approach with image processing techniques for diagnosis of retinal diseases. Multimed. Syst..

[74] Arabahmadi M., Farahbakhsh R., Rezazadeh J. (2022). Deep learning for smart Healthcare—A survey on brain tumor detection from medical imaging. Sensors.

[75] Salehi A.W., Khan S., Gupta G., Alabduallah B.I., Almjally A., Alsolai H., Mellit A. (2023). A study of CNN and transfer learning in medical imaging: Advantages, challenges, future scope. Sustainability.

[76] Jabbar A., Naseem S., Mahmood T., Saba T., Alamri F.S., Rehman A. (2023). Brain tumor detection and multi-grade segmentation through hybrid caps-VGGNet model. IEEE Access.

[77] Celebi A.R.C., Bulut E., Sezer A. (2023). Artificial intelligence based detection of age-related macular degeneration using optical coherence tomography with unique image preprocessing. Eur. J. Ophthalmol..

[78] Reis H.C., Turk V. (2022). COVID-DSNet: A novel deep convolutional neural network for detection of coronavirus (SARS-CoV-2) cases from CT and Chest X-Ray images. Artif. Intell. Med..

[79] Kaur P., Harnal S., Tiwari R., Alharithi F.S., Almulihi A.H., Noya I.D., Goyal N. (2021). A hybrid convolutional neural network model for diagnosis of COVID-19 using chest X-ray images. Int. J. Environ. Res. Public Health.

[80] Swaraj K.K., Kiruthiga G., Madhu K.P. (2021). Detection of liver cancer from CT images using CAPSNET. ICTACT J. Image Video Process..

[81] Wang Q., Chen A., Xue Y. (2023). Liver ct image recognition method based on capsule network. Information.

[82] Iyyanar G., Gunasekaran K., George M. (2024). Hybrid Approach for Effective Segmentation and Classification of Glaucoma Disease Using UNet++ and CapsNet. Rev. d’Intell. Artif..

[83] Kalyani G., Janakiramaiah B., Karuna A., Prasad L.N. (2023). Diabetic retinopathy detection and classification using capsule networks. Complex Intell. Syst..

[84] Mascarenhas M., Ribeiro T., Afonso J., Ferreira J.P., Cardoso H., Andrade P., Macedo G. (2022). Deep learning and colon capsule endoscopy: Automatic detection of blood and colonic mucosal lesions using a convolutional neural network. Endosc. Int. Open.

[85] Afriyie Y., Weyori B.A., Opoku A.A. (2022). Gastrointestinal tract disease recognition based on denoising capsule network. Cogent Eng..

[86] Hasnain M.A., Ali S., Malik H., Irfan M., Maqbool M.S. (2023). Deep learning-based classification of dental disease using X-rays. J. Comput. Biomed. Inform..

[87] AlSayyed A., Taqateq A., Al-Sayyed R., Suleiman D., Shukri S., Alhenawi E., Albsheish A. (2023). Employing CNN ensemble models in classifying dental caries using oral photographs. Int. J. Data Netw. Sci..

[88] Adla D., Reddy G.V.R., Nayak P., Karuna G. (2022). Deep learning-based computer aided diagnosis model for skin cancer detection and classification. Distrib. Parallel Databases.

[89] U.S. Food and Drug Administration (FDA) 510(k) Premarket Notification: Transpara (K181704). https://www.accessdata.fda.gov/scripts/cdrh/cfdocs/cfpmn/pmn.cfm?ID=K181704.

[90] U.S. Food and Drug Administration (FDA) 510(k) Premarket Notification: BriefCase (K203508), Aidoc Medical, Ltd. https://www.accessdata.fda.gov/scripts/cdrh/cfdocs/cfpmn/pmn.cfm?ID=K203508.

